# 679. Infective Endocarditis and Septic Emboli-Related Complications; Epidemiology and Impacts on Hospital Outcomes

**DOI:** 10.1093/ofid/ofab466.876

**Published:** 2021-12-04

**Authors:** Hansang Park, Monil Majmundar, Soumyajit Roy, Maria Rosa Velasquez-Espiritu, Kuldeep Ghosh, Khatuna Kadeishvili, Shobhana Chaudhari, Eliana A Lopez

**Affiliations:** 1 New York Medical College Metropolitan Hospital Center, Fort Lee, New Jersey; 2 New York Medical College Metropolitan Hospital, New York, New York; 3 NYCHHC Metropolitan NYMC, New York, New York

## Abstract

**Background:**

Infectious endocarditis (IE) remains a disease of high mortality, complications and a severe burden to the healthcare system despite advances in diagnostic techniques and treatments. There are several investigations of IE using a nation-based population cohort, however, with limited focus on septic emboli-related complications.

Figure 1. Flowchart of the study cohort. IE=Infective Endocarditis

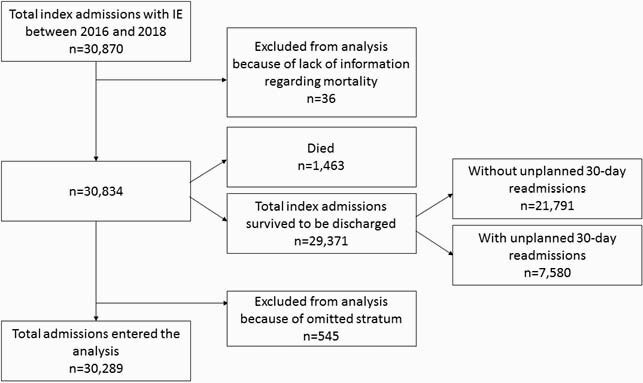

**Methods:**

We used the 2016 to 2018 National Readmission Database (NRD) to identify a primary diagnosis of admissions among adults (Age≥18) with IE. International Statistical Classification (ICD-10) codes were used to identify patients with a primary diagnosis of IE who experienced in-hospital septic emboli-related complications. Primary outcomes were mortality, length of stay, total cost and 30-day all-cause readmission. Uni- and Multivariate Linear, Logistic and Cox regression were used to assess statistical significance and a two-sided p-value less than 0.05 was considered significant.

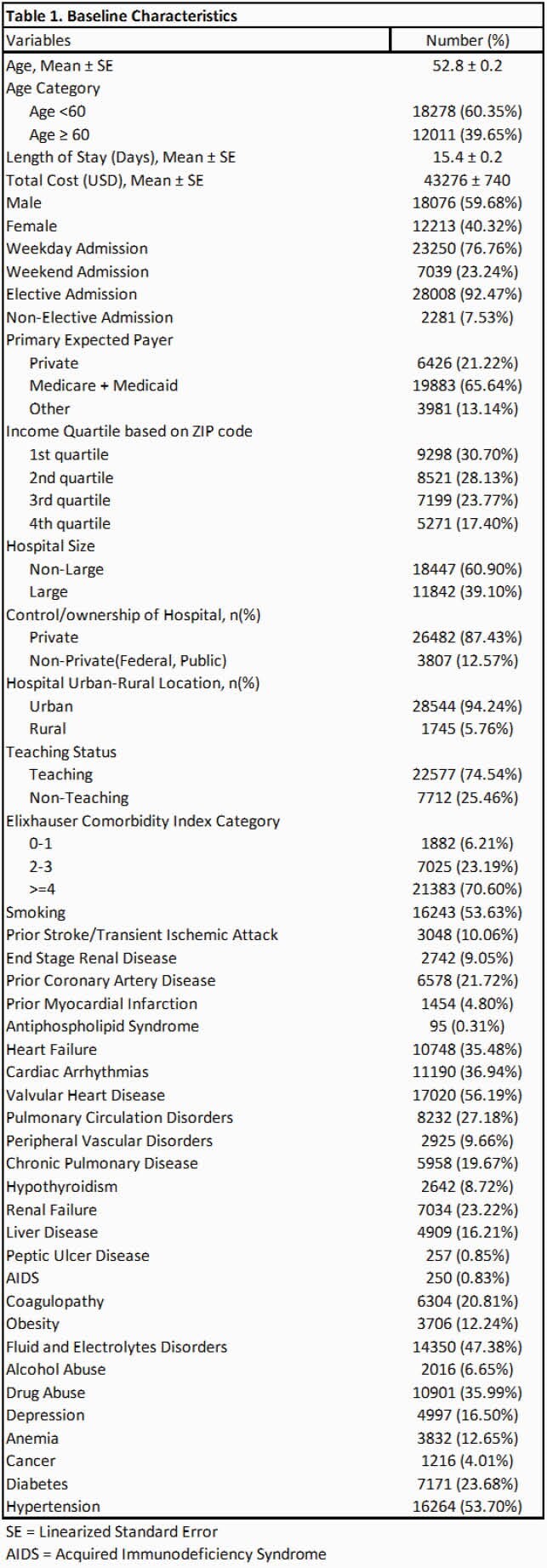

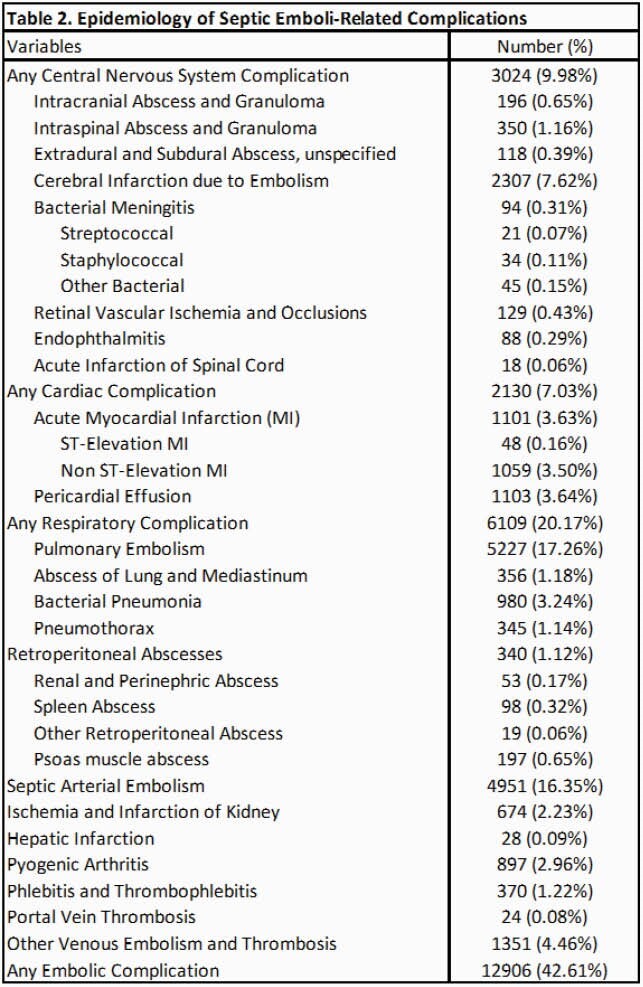

**Results:**

A total of 30,870 patients were admitted with a primary diagnosis of IE from 2016 to 2018 (December admissions were omitted). After excluding the patients with omitted information, 30,289 patients went into analysis. Baseline characteristics are shown in Table 1. Septic emboli-related complications were seen in 42.6% of the patients; about 10% had central nervous system (CNS) complications, 7% had cardiac complications and 20.2% had respiratory complications. Embolic complications of any kind were associated with higher mortality (Odds Ratio = 2.11 [1.74 – 2.54]), a longer length of stay (5.72 days [5.17 – 6.27]) and higher total costs (21,812 dollars [19,856 – 23,769]) while adjusted for baseline characteristics. Multivariate Cox regression to assess the risk of 30-day readmission was not statistically significant. Predictors of 30-day all-cause readmission among baseline characteristics and subgroups of embolic complications are shown in table 4 and table 5, respectively.

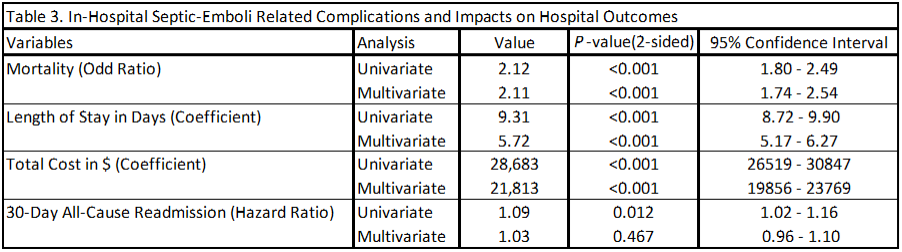

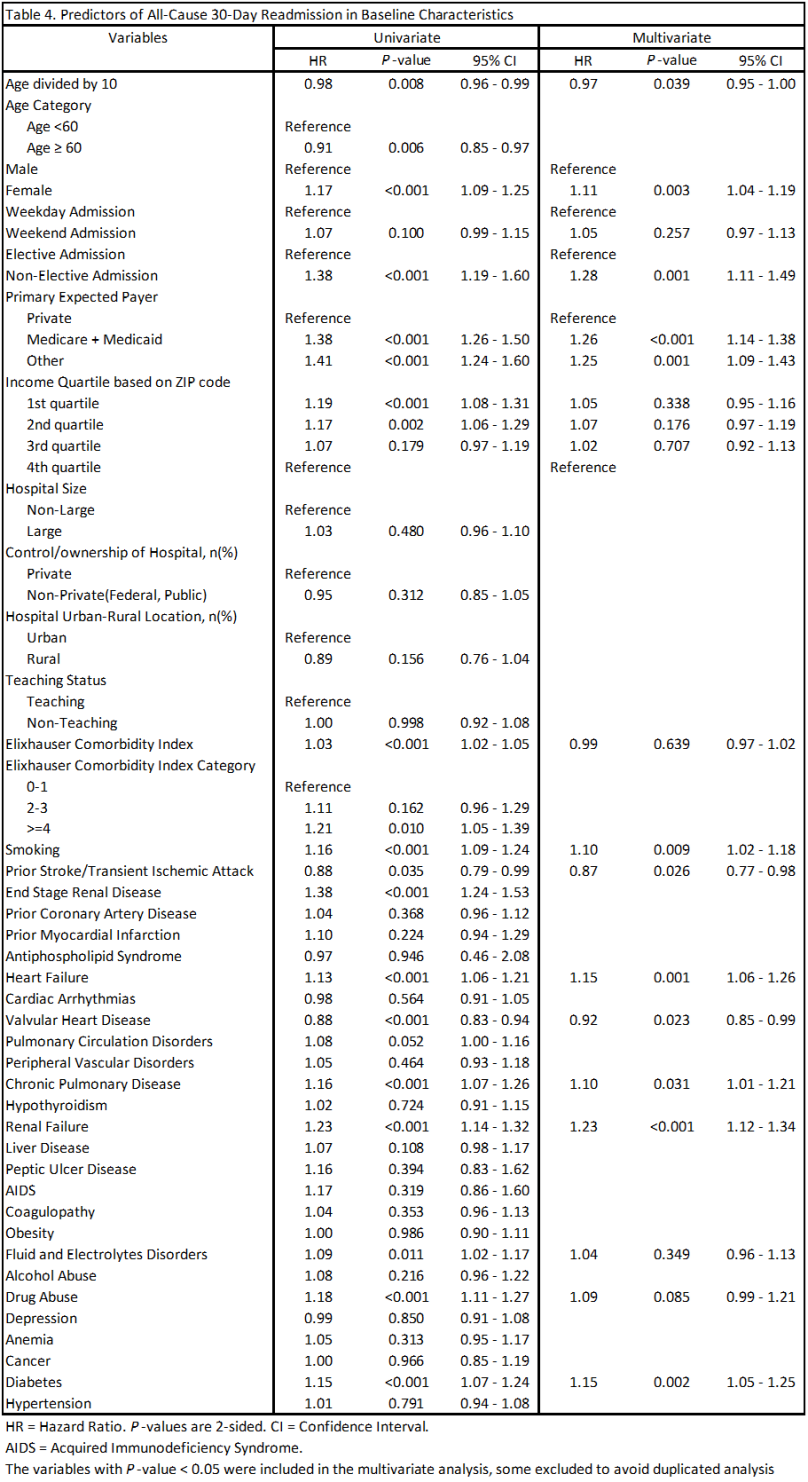

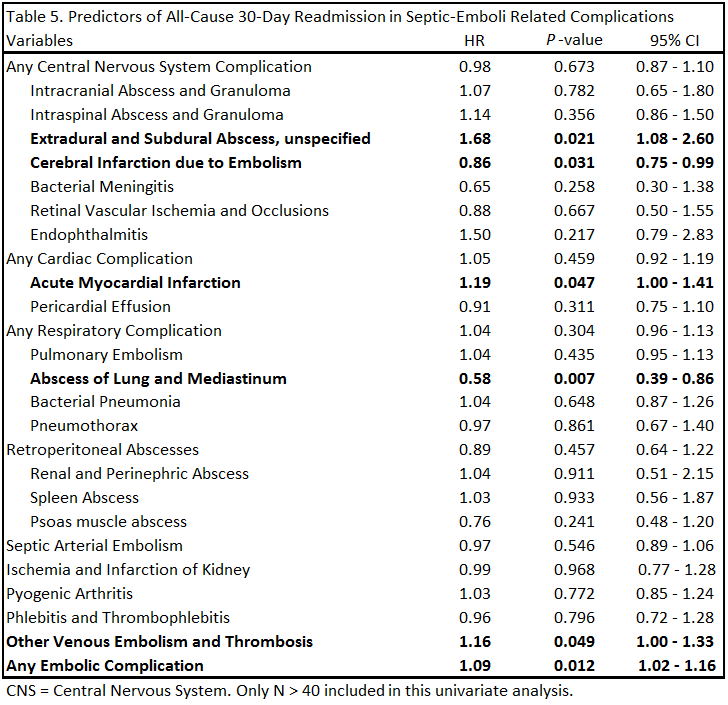

**Conclusion:**

The prevalence of septic emboli-related complications was up to 42.6% of patients admitted with the primary diagnosis of IE. These complications significantly impact hospital outcomes; including mortality, length of stay and total cost. Further studies are required to clarify the relationship between 30-day all-cause readmissions and embolic complications.

**Disclosures:**

**All Authors**: No reported disclosures

